# Modulation of Obesity-Related Lipid Metabolism by Sulforaphane Through AMPK-Centered Molecular Networks

**DOI:** 10.3390/ijms27167138

**Published:** 2026-08-09

**Authors:** Uyory Choe

**Affiliations:** Department of Food and Nutrition, College of Biomedical and Health Science, Konkuk University, 268 Chungwon-daero, Chungju-si 27478, Republic of Korea; foodtech@kku.ac.kr; Tel.: +82-43-840-3581

**Keywords:** sulforaphane, AMPK signaling pathway, lipid metabolism, obesity, cruciferous vegetables

## Abstract

Sulforaphane (SFN), an isothiocyanate produced from glucoraphanin in cruciferous vegetables, has been associated with the regulation of lipid storage, fatty acid oxidation, and autophagic lipid turnover. Therefore, in the current narrative review, AMPK-centered mechanisms were critically evaluated by distinguishing pathway association from experimentally demonstrated AMPK dependence. In cell and animal models, SFN treatment was associated with ACC phosphorylation, inhibition of mTORC1-SREBP signaling, CPT-1-related fatty acid oxidation, PGC-1α/PPAR-α transcriptional regulation, and ULK1-mediated lipophagy. However, most studies measured AMPK phosphorylation without pharmacological or genetic suppression, and one adipocyte study showed decreased AMPK phosphorylation during SFN-induced lipolysis. These findings indicate that SFN signaling can vary depending on the model, dose, and measured endpoint. Human studies have also shown highly variable SFN exposure and limited or inconsistent effects on lipid and glycemic outcomes, but AMPK-mediated lipid remodeling has not been directly demonstrated in target tissues. In addition, food matrix, myrosinase activity, dose, gut microbiota, and SFN-NIT formation can influence systemic exposure. Therefore, AMPK may be considered an important component of a broader SFN-responsive network rather than an exclusive mechanism. Further standardized human studies are required to evaluate quantified SFN exposure, lipid-related outcomes, tissue-relevant pathway biomarkers, and long-term safety.

## 1. Introduction

Cruciferous vegetables such as broccoli, Brussels sprouts, kale, and cabbages belong to the Brassicaceae family and possess a rich reservoir of complex, sulfur-containing compounds derived from amino acids known as glucosinolates (GLSs). Within the plant matrix, GLSs play a critical role in defense mechanisms against pests, pathogens, and herbivores [1]. Upon physical tissue disruption, such as through mastication, the enzyme myrosinase, ordinarily sequestered within tissue cells, is released to chemically hydrolyze GLSs into various degradation products, including isothiocyanates (ITCs) and nitriles (NITs) [1]. Among these, ITCs exhibit strong, pungent flavors and odors that act as chemical deterrents or toxins against herbivores. Concurrently, in humans, ITCs function as potent bioactive compounds capable of promoting overall well-being and preventing chronic metabolic disorders [2]. Over the past several decades, the metabolic pathways of GLSs and their derivatives have been widely investigated, establishing a robust correlation between the regular consumption of cruciferous vegetables and the prevention of chronic metabolic diseases via ITC activity [3].

Among the identified ITCs, SFN has been the most extensively investigated compound. Early studies mainly focused on its antioxidant, anti-inflammatory, and cytoprotective properties, while subsequent studies suggested that SFN can regulate metabolic signaling [4]. As a result, SFN has received increasing attention as a dietary compound for the regulation of lipid homeostasis. However, the strength of the available evidence varies considerably among cell, animal, and human studies. Recent global analyses have shown that the prevalence of obesity continues to increase across different regions and age groups [5,6]. Obesity is characterized by excessive lipid storage, ectopic lipid deposition, and reduced metabolic flexibility. Therefore, pathways involved in lipid synthesis, oxidation, and organelle turnover have become important therapeutic targets. In the matter of energy regulation, AMPK is of particular interest, although SFN can also affect Nrf2, SIRT1, ERK/Akt, cell-cycle, inflammatory, and autophagic pathways. Accordingly, the metabolic effects of SFN should be understood as pleiotropic and dependent on the experimental condition rather than as a result of one universally activated pathway [4,7].

The systemic conversion of GLSs into active ITCs proceeds through two primary routes: hydrolysis by plant-intrinsic myrosinase or degradation via the gut microbiota. In intact plants, GLSs like glucoraphanin reside in mesophyll cells, whereas myrosinase is isolated within specialized myrosin cells. Hence, mechanical cellular damage is required to facilitate enzyme-substrate contact and generate SFN [8]. However, mature cruciferous vegetables (e.g., mature broccoli) are typically subjected to thermal processing prior to ingestion. Cooking routinely denatures the heat-labile plant myrosinase, profoundly restricting the primary hydrolysis of GLSs in the pre-ingestive phase. Crucially, intact glucosinolates cannot be distributed to peripheral tissues from blood circulation and are excreted in the urine or feces even if they traverse enterocytes; thus, complete enzymatic hydrolysis into active ITCs is important for systemic efficacy [9]. While intact GLSs reaching the large intestine can undergo secondary hydrolysis via the myrosinase-like activity of certain bacterial strains, this process occurs exclusively in the distal colon, and its metabolic contribution remains highly variable due to pronounced inter-individual differences in gut microbiota profiles [10].

Microgreens and sprouts have been suggested as useful food matrices for preserving glucosinolate–myrosinase interactions because they are commonly consumed raw or with minimal processing [11]. Their potential advantage is not a guaranteed therapeutic effect, but an increased possibility of producing SFN before or during gastrointestinal digestion when active myrosinase is retained. However, SFN yield can be affected by cultivar, growing condition, storage, processing, mastication, and meal composition. Therefore, the amount of plant material administered should not be considered equivalent to the amount of SFN systemically available.

Although previous reviews have summarized the antioxidant, anti-inflammatory, and chemopreventive properties of SFN, limited information is available from a critical perspective on lipid-metabolic pathways and the strength of evidence supporting AMPK causality.

Therefore, the current review aims at understanding how SFN is associated with the regulation of lipogenesis, mitochondrial fatty acid oxidation, PGC-1α/PPAR-α-dependent transcription, and autophagic lipid turnover. In particular, the cited studies were examined to determine whether they only reported AMPK phosphorylation or directly evaluated the requirement for AMPK using pharmacological inhibition or genetic suppression.

In addition to the molecular mechanisms, bioavailability, gut microbial metabolism, food processing, dose translation, human clinical evidence, and safety were considered. This approach may help distinguish mechanistic plausibility from demonstrated efficacy and identify the evidence required before SFN can be considered a predictable intervention for obesity-related lipid disorders.

### Literature Review Approach and Evidence Appraisal

Because the available evidence includes mechanistic cell studies, heterogeneous animal models, food-matrix interventions, and human trials with different outcomes, the current review was conducted as a critical narrative synthesis rather than a meta-analysis. A focused literature update through July 2026 was performed using PubMed and reference-list screening with combinations of “sulforaphane,” “glucoraphanin,” “AMPK,” “lipid metabolism,” “adipocyte,” “fatty acid oxidation,” “lipophagy,” “clinical trial,” “microbiome,” “dose,” and “safety.” Primary studies were preferentially selected, and representative evidence was evaluated according to the model, exposure, duration, sample size when available, metabolic endpoint, use of AMPK perturbation, and translational limitation.

Pooled effect sizes were not calculated because the included studies differed substantially in intervention form, dose unit, exposure duration, tissue, disease condition, and endpoint definition. In addition, many mechanistic studies reported selected immunoblot fold changes without complete variance and group-level data, which limited comparable quantitative synthesis. Therefore, Table 1 presents a structured evidence map rather than a pooled estimate and identifies studies in which causal AMPK testing, replication, or human validation was not available.

## 2. Glucoraphanin Conversion and Systemic Bioavailability

### 2.1. Systemic ADME Profiles of Intact Glucosinolates Versus Isothiocyanates

The biological effects of cruciferous foods are partially determined by the different absorption, distribution, metabolism, and excretion profiles of intact GLSs and their hydrolysis products. Parent GLSs that are not hydrolyzed by plant myrosinase can pass through the upper gastrointestinal tract and may be absorbed or delivered to the colon for microbial metabolism [10]. Intact GLSs generally have lower cellular permeability than ITCs. However, they should not be described as completely inactive or unable to cross biological barriers because their absorption and disposition can vary depending on the compound and study design [23].

On the other hand, SFN is rapidly conjugated through the mercapturic acid pathway, and SFN-derived metabolites have been detected in plasma, urine, and tissues after the consumption of appropriate food matrices or extracts [24,25]. Systemic exposure can be greatly affected by the delivered chemical form, activity of myrosinase, food processing, and individual metabolism. Therefore, the detection of SFN in tissues alone does not indicate a clinically meaningful metabolic effect.

### 2.2. Microbial Hydrolysis Bypass via Gut Microbiota

**Figure 1:**
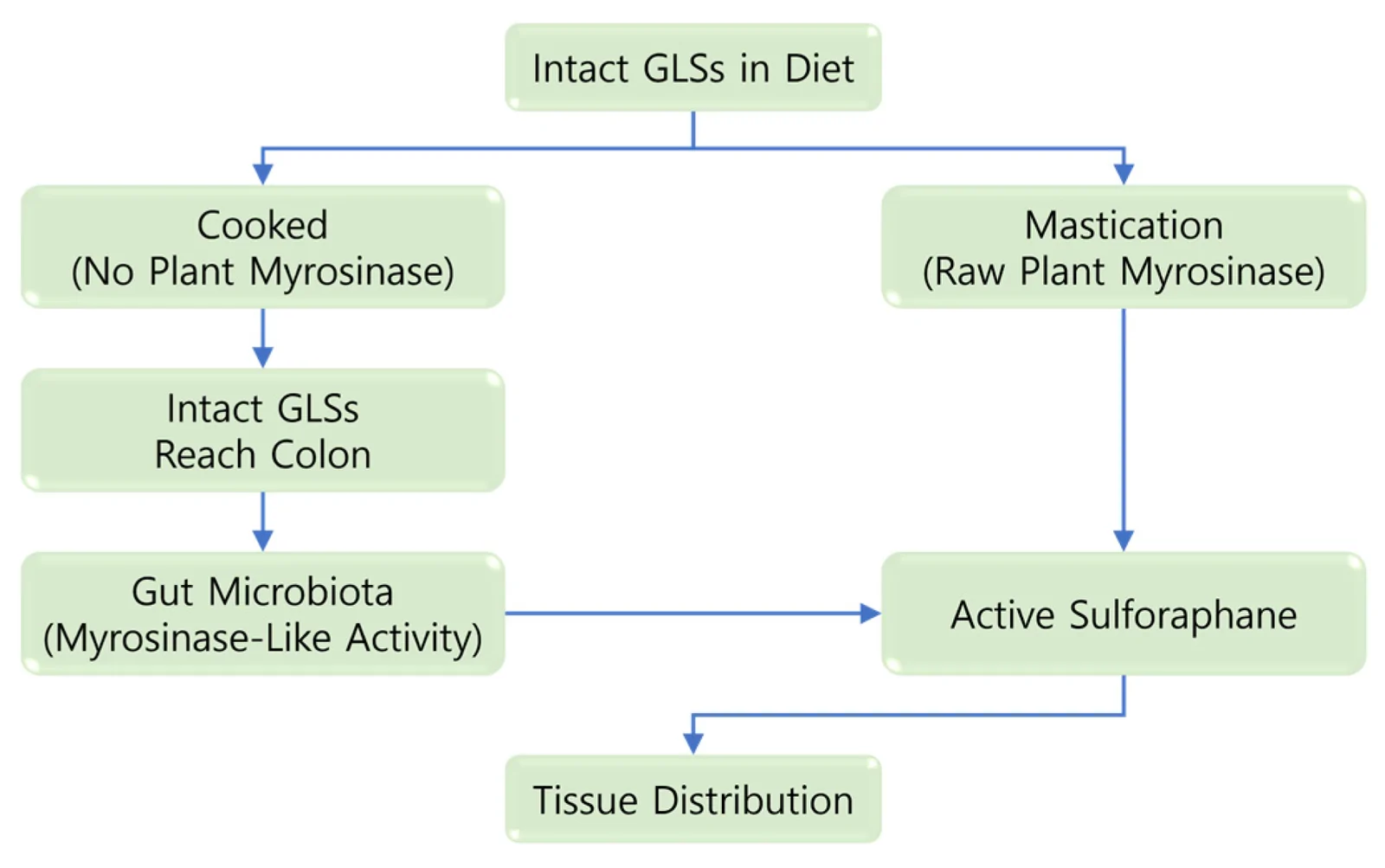
Schematic representation of dietary glucoraphanin bio-conversion pathways into active sulforaphane.

When plant myrosinase is absent or inactivated, intact GLSs reaching the large intestine can be hydrolyzed by bacterial enzymes having myrosinase-like activity [26]. As shown in Figure 1, this microbial pathway may contribute to ITC formation. However, its efficiency and product profile can vary considerably among individuals [27,28].

Dietary glucoraphanin can be hydrolyzed before absorption by plant myrosinase retained in raw or minimally processed foods. When the enzyme is inactivated by thermal processing, a portion of intact glucoraphanin may reach the colon and be converted by the gut microbiota. Both pathways can produce SFN; however, the relative yield can vary depending on processing, food matrix, gastrointestinal transit, and microbiome function. Therefore, the two pathways should not be expected to produce the same systemic exposure.

Microbial hydrolysis in the large intestine can contribute to the formation of SFN metabolites, but the amount distributed to peripheral tissues and its relevance to specific metabolic outcomes are not fully understood [10]. Inter-individual differences in microbiome composition and function have been associated with variations in SFN and SFN-NIT production [29,30]. However, these associations mainly reflect bioavailability and metabolite formation and should not be directly interpreted as anti-obesity efficacy.

## 3. Regulation of Fatty Acid Synthesis and Mitochondrial β-Oxidation

### 3.1. SFN-Associated AMPK Activation and Its Upstream Regulation

AMPK is a major energy-sensing kinase that regulates anabolic and catabolic processes [31,32]. Several studies have shown that SFN treatment increased the phosphorylation of AMPK and its downstream target ACC in HFD-fed mice and other disease-specific models [14,33,34]. These results support the involvement of the pathway. However, increased phosphorylation after SFN treatment does not necessarily demonstrate that AMPK is required for the metabolic response. It is worth noting that most studies did not combine pathway measurements with AMPK knockout, isoform-specific silencing, or validated rescue experiments.

The interpretation of the available evidence also requires consideration of different upstream kinases and contradictory responses. LKB1 is a well-established upstream kinase of AMPK and has been associated with SFN-responsive disease models [33,34]. In addition, Ca2+/calmodulin-dependent protein kinase kinase β (CaMKKβ) can activate AMPK without changes in the AMP/ATP ratio [35,36], although its direct involvement in SFN-mediated lipid regulation has not been demonstrated. A recent study using HFD-fed mice showed that Compound C attenuated the effects of both an SFN-rich broccoli seed hydrolysate and SFN, providing stronger pharmacological evidence for the involvement of AMPK [17]. However, Compound C is not specific to AMPK, and genetic confirmation is still required. On the contrary, SFN-induced lipolysis in mature adipocytes was accompanied by decreased AMPK phosphorylation [13]. Therefore, AMPK should be considered a signaling node that responds differently depending on the experimental condition rather than a universally required switch.

### 3.2. Inhibition of Fatty Acid Synthesis via the ACC Pathway

When activated, AMPK can phosphorylate ACC and decrease malonyl-CoA formation, thereby suppressing a rate-limiting step of de novo fatty acid synthesis [15,37]. In SFN-treated models, increased phosphorylation of AMPK and ACC was observed together with decreased lipid accumulation. Nonetheless, these parallel changes should be interpreted as mechanistically consistent rather than causal unless AMPK dependence is directly demonstrated.

In models where SFN increases AMPK activity, ACC phosphorylation can decrease the conversion of acetyl-CoA to malonyl-CoA. The reduced malonyl-CoA level may relieve the inhibition of CPT-1 and facilitate the mitochondrial transport of long-chain fatty acyl groups. The figure presents a biologically plausible AMPK-centered mechanism, but does not indicate that every step has been causally demonstrated in all SFN models.

**Figure 2:**
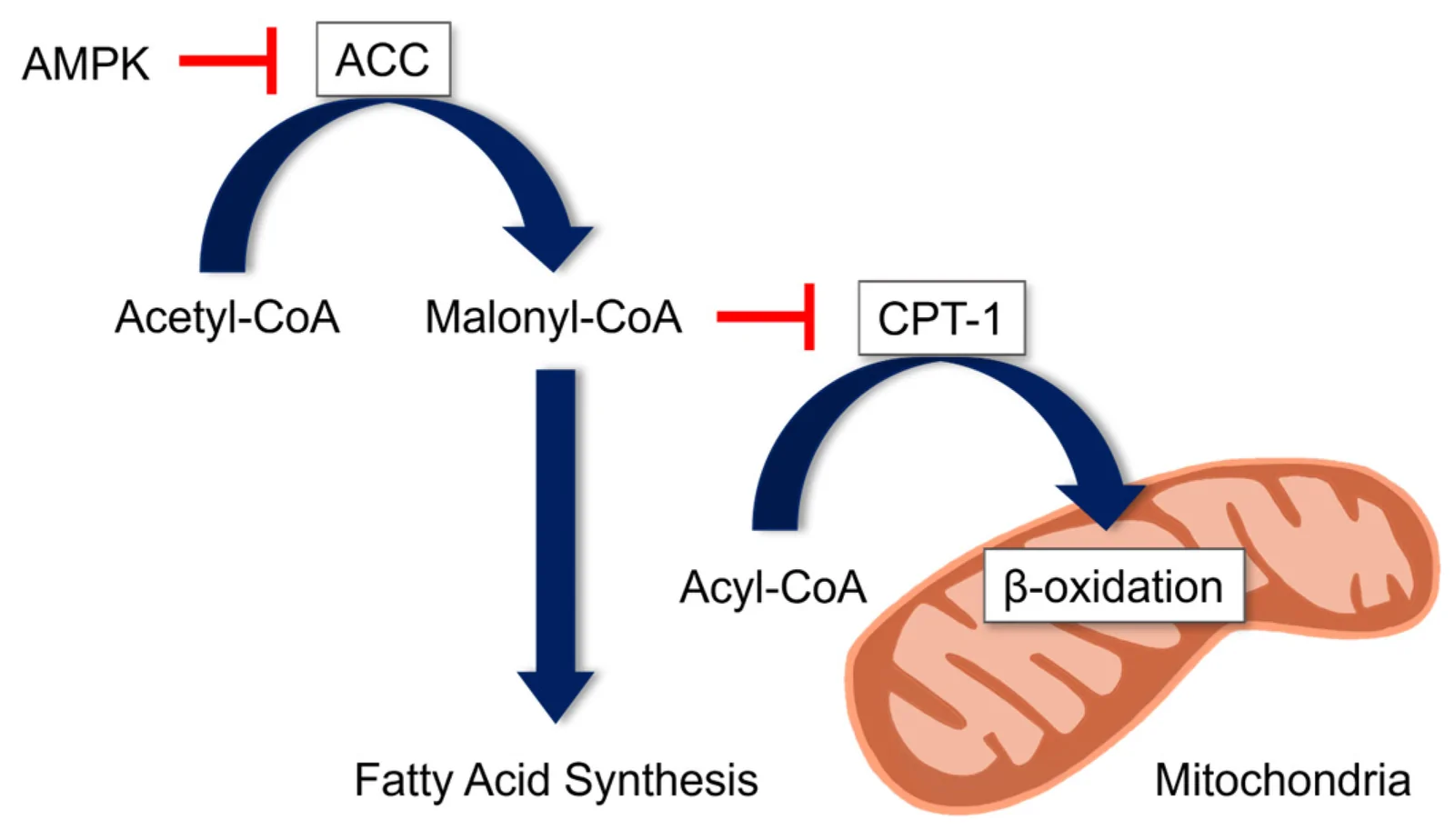
Proposed AMPK-centered pathway by which SFN regulates ACC inhibition and mitochondrial fatty acid oxidation.

As shown in Figure 2, ACC phosphorylation may connect AMPK activation with decreased lipogenesis. In several models, SFN-associated increases in AMPK and ACC phosphorylation were accompanied by lower lipid accumulation in adipose tissue or the liver [15,33,38]. But the possible contributions of Nrf2, transcriptional inhibition of adipogenesis, altered food intake, and other pathways cannot be excluded.

### 3.3. Potential Enhancement of Mitochondrial β-Oxidation Through CPT-1

A decrease in malonyl-CoA can enhance mitochondrial fatty acid β-oxidation by relieving the inhibition of CPT-1. During β-oxidation, fatty acyl-CoA is progressively converted into acetyl-CoA for energy production [13,39]. However, direct measurements of metabolic flux are rarely performed in SFN studies. Therefore, changes in oxidative genes or proteins should not be considered equivalent to a directly measured oxidation rate.

CPT-1 regulates the transport of long-chain fatty acyl groups into the mitochondrial matrix and is inhibited by malonyl-CoA [40,41]. Thus, SFN-associated changes in ACC activity and malonyl-CoA provide a possible mechanism for increasing fatty acid oxidation. However, the magnitude, tissue specificity, and AMPK dependence of this response remain unclear because previous studies used different models, doses, exposure periods, and surrogate endpoints.

### 3.4. Dose–Response, Treatment Duration, and Pleiotropic Signaling

SFN has been used at low micromolar concentrations for several hours or at higher concentrations for several days in cell studies, whereas animal studies have used dietary percentages, extracts, or milligram-per-kilogram doses. These experimental designs cannot be directly compared. In connection with adipocyte responses, the results also differed depending on differentiation stage and measured endpoint. For example, 2.5–10 μM SFN increased lipolysis with decreased AMPK phosphorylation [13], whereas 10 μM SFN increased AMPK-mTOR-ULK1-associated lipophagy in another model [16]. In addition, prolonged exposure to 100 μM SFN decreased adipocyte viability [16]. The current results suggest that signaling observed at high concentrations may be caused by cellular stress rather than a therapeutically useful response.

SFN can also activate or regulate KEAP1-Nrf2, SIRT1, ERK/Akt, p27-cell-cycle, inflammatory, and autophagic pathways [4,12,24,37,42]. These pathways can interact with AMPK or independently influence lipid-related phenotypes. Therefore, concentration-response and time-course experiments, direct AMPK perturbation, evaluation of cell viability, and parallel assessment of alternative pathways are required before a specific effect is attributed to AMPK.

## 4. Transcriptional Regulation of Lipid Catabolism by PGC-1α and PPAR-α

### 4.1. AMPK-Associated Regulation of PGC-1α

AMPK can regulate long-term oxidative metabolism through PGC-1α, a transcriptional coactivator involved in mitochondrial biogenesis and fatty acid oxidation [37]. SFN treatment has been reported to alter the expression or activation of PGC-1α. Yet PGC-1α can also be regulated by SIRT1, redox signaling, and other kinases. Hence, the AMPK-PGC-1α pathway may explain the response in selected models, but should not be considered the only route by which SFN regulates mitochondrial programs [39].

### 4.2. Nuclear Coactivation of PPAR-α by PGC-1α

PGC-1α acts as a coactivator of nuclear receptors including PPAR-α. PPAR-α is abundantly expressed in the liver and regulates genes associated with fatty acid transport and mitochondrial or peroxisomal oxidation [43,44]. It is worth noting that PGC-1α does not form a stable cytoplasmic complex that subsequently translocates to the nucleus, as previously depicted. Rather, PGC-1α coactivates DNA-bound nuclear PPAR-α and the associated transcriptional machinery.

SFN-responsive signaling may increase the amount or activity of PGC-1α. Within the nucleus, PGC-1α can coactivate PPAR-α at peroxisome proliferator response elements (PPREs) and increase the transcription of genes involved in fatty acid transport and oxidation, including ACOX1, FABP, MCAD, LCAD, and CPT-1. In the figure, AMPK is presented as one contributing signaling node rather than an obligatory upstream event in all models.

**Figure 3:**
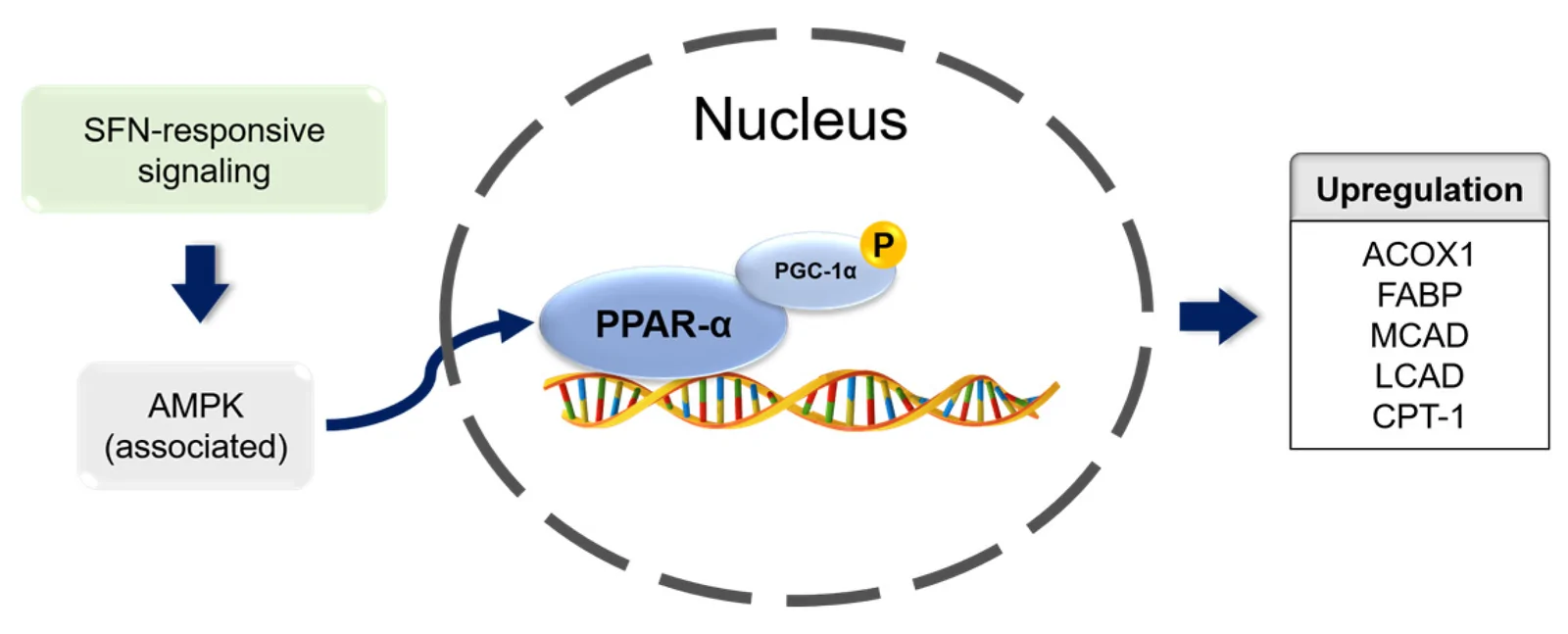
Proposed AMPK-associated transcriptional regulation through PGC-1α and nuclear PPAR-α.

As depicted in Figure 3, the relevant transcriptional regulation occurs in the nucleus, where PGC-1α increases PPAR-α-dependent gene expression [45,46]. Evidence for SFN-mediated activation of this pathway is mainly based on changes in pathway markers and gene expression. Even so, direct evidence that AMPK is required for PGC-1α/PPAR-α activation remains limited.

### 4.3. Nuclear PPRE Binding and Upregulation of Downstream Metabolic Genes

At PPRE-containing regulatory regions, PPAR-α and its coactivators can increase the transcription of enzymes involved in fatty acid transport and mitochondrial or peroxisomal oxidation [47]. In SFN studies, these transcriptional changes support a possible increase in lipid catabolism. Nevertheless, their functional significance should be further confirmed using substrate oxidation or metabolic-flux measurements.

## 5. Inhibition of Lipogenesis and Activation of Lipophagy

### 5.1. AMPK-Mediated Suppression of the mTORC1 Anabolic Master Regulator

mTORC1 is a nutrient-responsive regulator involved in cell growth, protein synthesis, and lipid biosynthesis [48]. AMPK can inhibit mTORC1 through the phosphorylation of upstream and complex-associated targets, thereby connecting cellular energy status with the suppression of anabolic metabolism. Although SFN-associated changes in this pathway have been reported, the relative contribution of AMPK and redox- or stress-responsive pathways can vary among models [49].

Activated AMPK can inhibit mTORC1, which may decrease SREBP processing and the expression of lipogenic enzymes including FAS, SCD, and ACC. AMPK can also facilitate ULK1 activation directly or indirectly through mTORC1 inhibition, thereby promoting the autophagic turnover of lipid droplets. These pathways are well-established components of cellular energy regulation. However, their complete causal sequence after SFN treatment has been demonstrated only in selected experimental models.

**Figure 4:**
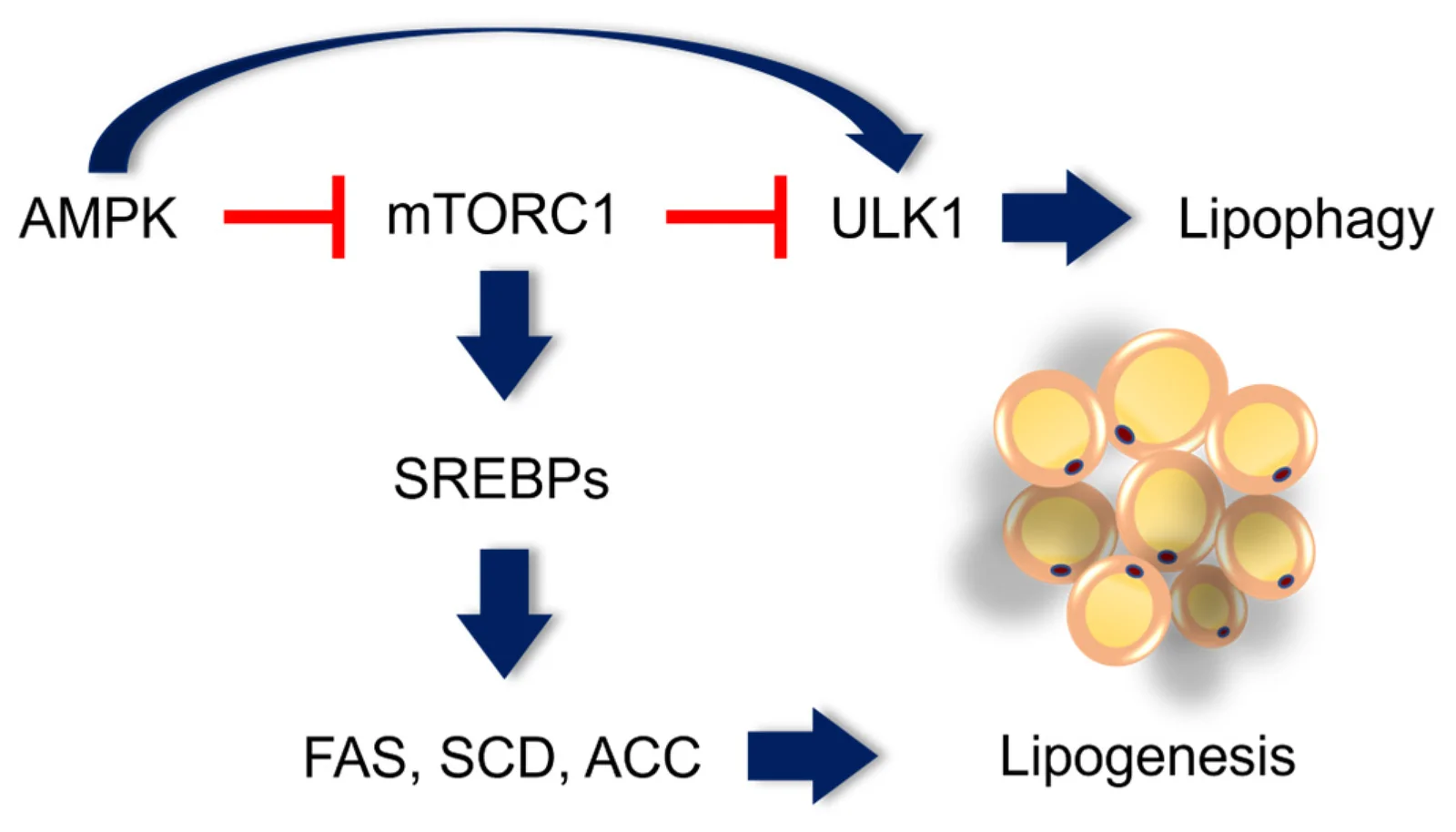
Proposed AMPK-mTORC1-SREBP and AMPK-ULK1 pathways by which SFN regulates lipogenesis and lipophagy.

As shown in Figure 4, AMPK-mediated inhibition of mTORC1 provides a possible connection between decreased lipid synthesis and increased autophagic turnover [50]. However, SFN can activate stress-responsive and antioxidant pathways independently of AMPK. Therefore, changes in mTORC1 should be interpreted within the broader signaling network.

### 5.2. Blunting Lipogenesis via the mTORC1-SREBP Transcriptional Axis

SREBPs regulate genes involved in the synthesis of cholesterol, fatty acids, and triglycerides [51]. Under obesogenic conditions, mTORC1 can facilitate SREBP processing and nuclear activity. Therefore, SFN-associated inhibition of this pathway may contribute to decreased lipogenesis. But previous studies differed in tissue, disease model, dose, and the measurement of SREBP processing, transcription, or downstream enzymes.

SFN treatment has been reported to decrease the abundance or activity of SREBP precursors in experimental models [52]. This result is consistent with the inhibition of the AMPK-mTORC1 pathway. Still, AMPK-independent regulation of SREBP stability, endoplasmic-reticulum processing, or transcription cannot be excluded.

### 5.3. Induction of ULK1-Mediated Lipophagy and Lipid Droplet Clearance

Lipophagy refers to the selective autophagic degradation of intracellular lipid droplets. ULK1 contributes to autophagosome initiation and can be activated by relief from mTORC1 inhibition and by direct phosphorylation through AMPK [53,54,55,56]. SFN-induced lipophagy has been investigated in hepatocyte and adipocyte models, suggesting that autophagic lipid turnover may occur together with changes in lipid storage.

Activated autophagic machinery can sequester components of lipid droplets and transport them to lysosomes, where triglycerides are hydrolyzed into fatty acids and glycerol [57]. Anyhow, whether this process increases net lipid oxidation, redistributes fatty acids, or becomes maladaptive can vary depending on the cellular condition and the balance among autophagic flux, mitochondrial capacity, and lipid supply.

Experimental studies have supported SFN-associated lipophagy in hepatocytes and mature 3T3-L1 adipocytes [16,42]. In the adipocyte study, mechanistic interventions provided stronger evidence for the involvement of autophagy. However, the results remain preclinical and do not demonstrate the reversal of human obesity or fatty liver disease. In addition, high concentrations and prolonged exposure can decrease cell viability. Therefore, a mechanistically active concentration range should be distinguished from cytotoxic stress.

## 6. Microbiome Inter-Individual Variation and SFN Bioactivity

### 6.1. Gut Microbiota Profiles and Inter-Individual Pharmacokinetic Variation

Systemic SFN exposure can vary greatly after the consumption of cruciferous foods or extracts [58,59]. When plant myrosinase is absent, gut microbial metabolism becomes more important. However, it is not the only determinant of exposure because food matrix, dose, gastrointestinal transit, host conjugation, and renal elimination can also contribute. Human studies have associated microbiome composition with variations in SFN and SFN-NIT metabolites [10,30].

Several bacterial taxa, including Roseburia, Bifidobacterium, Bacteroides, Ruminococcus, Dorea, Alistipes, and Blautia, have been associated with ex vivo or in vivo glucosinolate metabolism [30,60,61]. Still, these associative results do not demonstrate that a single taxon determines systemic SFN exposure or obesity-related outcomes. A randomized trial conducted in 2025 in individuals with prediabetes showed exploratory relationships among baseline microbial features, serum SFN concentration, and glycemic response [22]. It is worth noting that the prespecified primary endpoint was not achieved and lipid outcomes were not used for the responder analysis.

### 6.2. Formation of SFN-NIT and Its Uncertain Biological Significance

**Figure 5:**
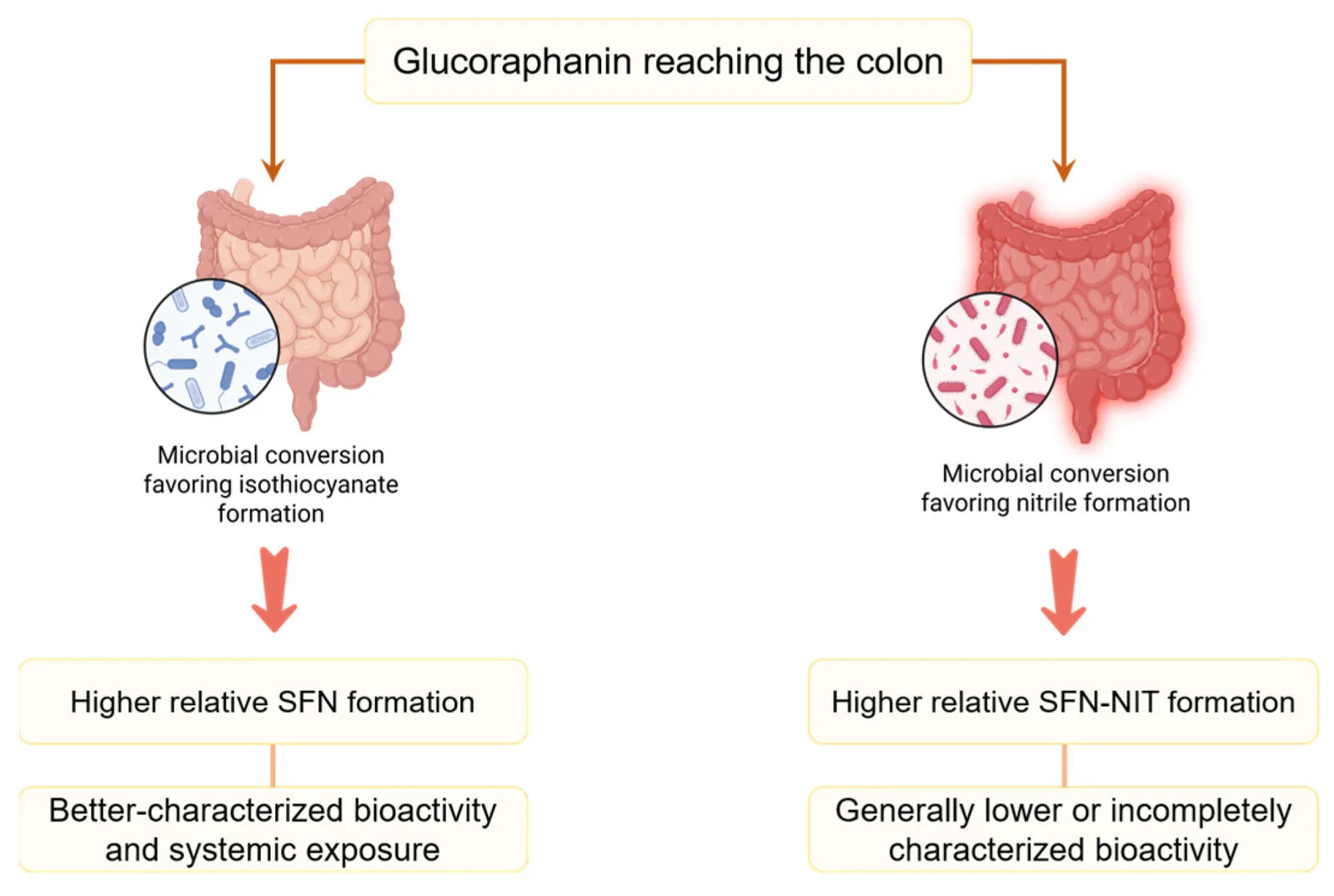
Proposed microbial conversion of glucoraphanin to SFN and SFN-NIT and the current limitations in biological interpretation. Created in BioRender. Choe, U. (2026); http://BioRender.com/axfxk8g (accessed on 28 July 2026).

Depending on enzymatic conditions and microbial function, glucoraphanin hydrolysis can produce SFN or alternative products including SFN-NIT [10,30]. As shown in Figure 5, this branching pathway may contribute to inter-individual variation in metabolite profiles. However, the relative production of either metabolite should not be considered a validated determinant of anti-obesity efficacy.

Dietary glucoraphanin reaching the colon can be converted by the gut microbiota through pathways favoring either isothiocyanate or nitrile formation. A greater relative production of SFN is expected to increase exposure to a metabolite with better-characterized molecular activity, whereas SFN-NIT has shown lower activity in the experimental assays evaluated to date. However, SFN-NIT should not be regarded as biologically inactive because its pharmacokinetics and biological effects have not been fully characterized. Therefore, the figure distinguishes metabolite formation from clinical consequences that have not yet been demonstrated.

Available studies have shown that SFN-NIT is less bioactive than SFN in several experimental assays, but its biological significance is still not fully understood [30,61]. Individuals producing a greater proportion of SFN-NIT may have lower exposure to SFN. However, there is currently no evidence that this metabolite profile directly predicts decreased adiposity, improved lipid profiles, or other clinical anti-obesity outcomes. Future studies should quantify both metabolites and prospectively relate them to predefined metabolic endpoints rather than assigning beneficial or adverse clinical phenotypes based only on microbiome composition.

### 6.3. Human Exposure and Its Translational Relevance

Human pharmacokinetic studies have shown that dietary or supplemental SFN can produce circulating and urinary metabolites, but the nominal dose does not represent systemic exposure. In healthy adults administered 200 μmol SFN/day, fresh broccoli sprouts produced approximately threefold higher plasma and urinary metabolite concentrations than a myrosinase-treated extract, while divided dosing prolonged exposure [21]. This variation makes it difficult to compare human exposure with cell studies using a fixed micromolar concentration for an extended period.

Clinical evidence for lipid-related outcomes is limited. In patients with type 2 diabetes, the consumption of 10 g/day broccoli sprout powder for 4 weeks decreased triglycerides and selected atherogenic indices [18]. Additionally, two 12-week dietary trials showed that 400 g/week high-glucoraphanin broccoli produced a modestly greater reduction in LDL-C than standard broccoli [19]. However, these interventions used complex food matrices and did not measure AMPK signaling in target tissues; therefore, the outcomes cannot be attributed solely to SFN. A 12-week intervention providing approximately 150 μmol SFN/day improved glycemic control mainly in participants with dysregulated diabetes [20], but did not demonstrate the AMPK-lipid pathways proposed from preclinical studies. A recent prediabetes trial also showed exploratory microbiome-response associations, although the prespecified primary fasting-glucose endpoint was not achieved and prediction of lipid responses was not established [22]. Taken together, dietary exposure to SFN can be biologically measurable and may affect selected metabolic endpoints, but direct human evidence for AMPK-mediated regulation of obesity-related lipid metabolism remains insufficient.

### 6.4. Dose Translation, Safety, and Potential Drug Interactions

Doses used in preclinical studies frequently exceed exposure from a normal diet. For example, previous studies used 0.1% SFN in the mouse diet for 6 weeks [14], acute or repeated milligram-per-kilogram doses [16,17], and sustained micromolar concentrations in cultured cells. The conversion of these regimens to a human-equivalent dose is uncertain because absorption, myrosinase activity, metabolism, and exposure kinetics differ depending on species and formulation. Human studies have used food- or extract-based interventions ranging from tens to hundreds of micromoles per day. However, these amounts generally require standardized sprouts or supplements rather than ordinary servings of mature cooked vegetables [20,21,62,63].

Short-term human studies have generally shown acceptable tolerability. A phase I trial administering broccoli sprout glucosinolate or isothiocyanate preparations every 8 h for 7 days found no consistent clinical or laboratory toxicity [62]. In addition, a beverage providing 600 μmol glucoraphanin and 40 μmol SFN daily was administered for 12 weeks to 291 adults [63], and an 84-day study in 45 women showed no adverse changes in thyroid function or autoimmunity [64]. These results are reassuring. But they do not establish long-term safety in individuals with obesity, polypharmacy, pregnancy, or chronic liver and kidney diseases. Potential drug interactions should also be directly evaluated rather than predicted only from in vitro assays. For example, 450 μmol SFN/day for 7 days did not antagonize rifampicin-induced human PXR activity in a randomized crossover trial [65], but this result does not exclude interactions involving other enzymes or transporters. Because a validated therapeutic window or upper intake level for chronic SFN supplementation has not been established, high-dose intake should be evaluated using exposure biomarkers, adverse-event monitoring, and medication review.

## 7. Conclusions and Future Perspectives

### 7.1. Synthesis of Multi-Targeted AMPK Metabolic Regulation

The current evidence supports an AMPK-centered mechanism of SFN action, but does not indicate that all effects are exclusively dependent on AMPK. In cell and animal models, SFN-associated changes in AMPK, ACC, mTORC1-SREBP, PGC-1α/PPAR-α, and ULK1 were consistent with decreased lipogenesis, increased fatty acid oxidation, and enhanced lipophagy. Nonetheless, the strength of causal evidence was not consistent among studies. Many studies reported phosphorylation or expression changes without suppressing AMPK, and one adipocyte model showed decreased AMPK phosphorylation during SFN-induced lipolysis [13]. In addition, a recent mouse study using Compound C provided stronger but still pharmacologically limited evidence for AMPK involvement [17].

Accordingly, the major conclusions of the current review are summarized according to the strength of the evidence rather than as therapeutic claims.

First, reduced lipogenesis may be explained by AMPK-dependent ACC phosphorylation and inhibition of the mTORC1-SREBP pathway. However, direct AMPK dependence has been demonstrated in only a limited number of models.

Second, decreased malonyl-CoA and increased expression of oxidative genes support the potential enhancement of fatty acid oxidation. Yet direct metabolic-flux measurements and validation in human tissues remain limited.

Third, SFN can increase the autophagic turnover of lipid droplets in preclinical models. But its efficacy against human obesity or metabolic liver disease has not been demonstrated.

### 7.2. Food Technology Innovations and Precision Nutritional Frameworks

Translation of these molecular findings requires standardized interventions and clinically relevant endpoints. Human studies should quantify administered glucoraphanin and SFN, myrosinase activity, circulating mercapturic-acid metabolites, adherence, and background cruciferous-vegetable intake. In addition, lipid profiles, ectopic fat, insulin sensitivity, and target-tissue or validated circulating pathway biomarkers should be evaluated.

Regarding food processing, preservation or addition of active myrosinase can increase the conversion of glucoraphanin to SFN [9,21,26,66]. Raw sprouts and microgreens are promising delivery matrices. However, their composition can vary and should not be assumed to provide a standardized dose. Therefore, controlled processing, chemical verification, and stability testing are required when these foods are used in mechanistic or clinical studies.

Concerning precision nutrition, microbiome information may help explain inter-individual variation in SFN exposure, as suggested by recent exploratory human data [22]. However, baseline microbiota cannot currently be considered a validated predictor of lipid or anti-obesity responses. Future randomized trials should prespecify microbiome hypotheses, validate the results in independent cohorts, and distinguish metabolite production from clinical efficacy. In addition, long-term safety, potential interactions, and the dose range that provides reproducible exposure without cytotoxic or pharmacological off-target effects require systematic evaluation. Until these data are available, SFN may be considered a promising dietary bioactive compound with substantial preclinical evidence but limited clinical validation for obesity-related lipid metabolism.

## Figures and Tables

**Table 1:** Representative experimental and clinical studies related to SFN-mediated lipid metabolism and AMPK-centered signaling.

SFN Exposure and Treatment Duration	Major Metabolic Findings	Evidence Appraisal and Study Limitations	Reference and Experimental Model
0–20 μM during early differentiation; 24–48 h at 20 μM	Decreased lipid accumulation and adipogenic transcription factors, together with p27-associated cell-cycle arrest	The results support a non-AMPK pathway involving cell-cycle and ERK/Akt signaling; however, the concentrations used in cells may exceed those achieved in tissues after dietary intake.	Choi et al. [12]/3T3-L1 preadipocytes
2.5–10 μM; 24 h	Increased HSL-dependent lipolysis, while AMPK Thr172 phosphorylation was decreased	It is worth noting that SFN-induced effects were not consistently associated with AMPK activation.	Lee et al. [13]/Mature 3T3-L1 adipocytes
0.1% SFN in diet; 6 weeks	Decreased body weight, adiposity, and hepatic triglyceride, together with increased AMPK and ACC phosphorylation	The results show pathway association without AMPK inhibition or knockout; in addition, the short study period and dietary dose cannot be directly compared with human intake.	Choi et al. [14]/HFD-fed mice
Broccoli sprout/mustard preparations; 12-week animal intervention	Decreased adipogenic markers and increased AMPK/ACC-associated signaling	The use of complex food extracts and small animal groups limits the attribution of the effects to SFN and the estimation of effect size.	Men et al. [15]/3T3-L1 cells and BPA-exposed mice
10 μM in cells with 0.5–9 h signaling assessment; 30 mg/kg acute mouse exposure	Increased autophagic flux and lipophagy through AMPK-mTOR-ULK1-associated signaling	The mechanistic autophagy experiments strengthen the interpretation; however, prolonged exposure to 100 μM decreased cell viability, and clinical relevance has not been evaluated.	Masuda et al. [16]/Mature adipocytes and mice
SFN 1–10 mg/kg or SFN-rich hydrolysate for 8 weeks; Compound C 5 mg/kg	Decreased adiposity and dyslipidemia, together with restored AMPK phosphorylation; the inhibitor attenuated these effects	The results provide pharmacological evidence for AMPK involvement; however, Compound C has off-target effects and the hydrolysate contains multiple constituents.	Jeon et al. [17]/HFD-fed mice
Broccoli sprout powder 5 or 10 g/day; 4 weeks	The 10 g/day treatment decreased triglycerides and atherogenic indices and increased HDL-C	The study was a short-term intervention using a whole-food powder; SFN exposure and activation of the AMPK pathway were not directly measured.	Bahadoran et al. [18]/Type 2 diabetes RCT
High-glucoraphanin or standard broccoli, 400 g/week; 12 weeks	High-glucoraphanin broccoli produced a modestly greater decrease in LDL-C	The food-matrix evidence was obtained from 130 participants; however, the outcome cannot be attributed only to SFN, and AMPK mediation cannot be inferred.	Armah et al. [19]/Two dietary RCTs
Broccoli sprout extract providing approximately 150 μmol SFN/day; 12 weeks	Improved glycemic control, mainly in participants with dysregulated diabetes; lipid effects were not the major findings	The results support metabolic translation but not AMPK-mediated lipid remodeling in humans; therefore, the subgroup findings should be interpreted with caution.	Axelsson et al. [20]/Type 2 diabetes RCT
Fresh sprouts or myrosinase-treated extract, 200 μmol SFN/day	Fresh sprouts produced approximately threefold higher plasma and urinary SFN metabolite levels	The results demonstrate that formulation and myrosinase activity affect exposure; however, obesity- or lipid-related efficacy was not evaluated.	Atwell et al. [21]/Healthy-adult pharmacokinetic study
Broccoli sprout extract once daily; 12 weeks (35 treatment, 39 placebo)	The primary fasting-glucose endpoint was not achieved; exploratory microbial features were associated with the response and SFN concentration	The study is relevant to personalized exposure; however, the findings are exploratory, are related to glycemia, and do not establish prediction of lipid or anti-obesity outcomes.	Dwibedi et al. [22]/Prediabetes RCT

Abbreviations: BPA, bisphenol A; HFD, high-fat diet; HSL, hormone-sensitive lipase; RCT, randomized controlled trial.

## Data Availability

No new data were created or analyzed in this study. Data sharing is not applicable to this article.

## Abbreviations

The following abbreviations are used in this manuscript:

ACC	Acetyl-CoA Carboxylase
ACOX1	Acyl-CoA Oxidase 1
AMPK	Adenosine Monophosphate-Activated Protein Kinase
CPT-1	Carnitine Palmitoyltransferase-1
FABP	Fatty Acid Binding Protein
FAS	Fatty Acid Synthase
GLSs	Glucosinolates
ITCs	Isothiocyanates
LCAD	Long Chain Acyl-CoA Dehydrogenase
LKB1	Liver kinase B1
MCAD	Medium Chain Acyl-CoA Dehydrogenase
mTORC1	Mammalian Target Of Rapamycin Complex 1
PGC-1α	Peroxisome Proliferator-Activated Receptor Gamma Coactivator 1-alpha
PPAR-α	Peroxisome Proliferator-Activated Receptor-alpha
SCD	Stearoyl-CoA Desaturase
SFN	Sulforaphane
SFN-NIT	Sulforaphane-Nitrile
SREBP	Sterol Regulatory Element-Binding Protein
ULK1	Unc-51-Like Autophagy-Activating Kinase 1
BPA	Bisphenol A
CaMKKβ	Ca2+/Calmodulin-Dependent Protein Kinase Kinase β
HFD	High-Fat Diet
HSL	Hormone-Sensitive Lipase
Nrf2	Nuclear Factor Erythroid 2-Related Factor 2
RCT	Randomized Controlled Trial
